# Supplementary material for: Calcineurin-mediated intracellular organelle calcium homeostasis is required for the survival of fungal pathogens upon extracellular calcium stimuli
Source: Virulence. 2021 Apr 12;12(1):1091–110. doi: 10.1080/21505594.2021.1909954 (PMC8043181; doi:10.1080/21505594.2021.1909954)
Supplement: Supplemental Material [file KVIR_A_1909954_SM4172.zip › supple dox.rtf]

Supplementary Material
Figure S1. Constructions of cnaA and cnaB deletion mutants in A. fumigatus via CRISPR-Cas9 system.
·	Schematic illustration of gRNA location and repair template for deleting genes via the CRISPR-Cas9 system. 
·	Details of gRNA and the repair template for cnaA and cnaB deletion. 
·	The colony morphologies of ÄcnaA, ÄcnaB, and wild-type A. fumigatus. 
·	Diagnostic PCR for ptrA integration in the corresponding gene's ORF. Each pair of diagnostic primers was complementary to upstream and downstream sequences flanking the deletion fragment.

Figure S2. Phenotypic characterization of the ÄcnaB mutant.
·	Differential interference contrast (DIC) images of hyphae grown in liquid MM with or without 10 mM CaCl2 at 37°C in stationary culture for 14 h. Scale bar represents 10 ìm. 
·	The morphology of mycelial pellets of the indicated strains grown in MM with or without 10 mM CaCl2 at 37°C for 60 h. 
·	Quantification of biomass production for the wild-type, ÄcnaB, and cnaBR complementary strains grown in MM with or without 10 mM CaCl2 at different time points (60, 72, 84, and 96 h).

Figure S3. mRNA expression analysis of pmcA and pmcB in the wild-type and ÄcnaA background strains and calcium-induced [Ca2+]vac transients in the wild-type, OE::pmcA, and OE::pmcB strains.
·	Transcript levels of pmcA/B in WT and OE::pmcA/B (pmcA/B-overexpressed strains) and in ÄcnaA and ÄcnaAOE::pmcA were determined by qRT-PCR. The indicated strains of A. fumigatus were incubated in MM for 36 h at 37°C. ✱✱p < 0.01.
·	Comparison of resting and dynamic [Ca2+]vac levels in the wild-type and OE::pmcA/B strains. Statistical significance was determined by Student's t-test. ✱p < 0.05; ✱✱p < 0.01.

Figure S4. Vacuolar morphology and GFP-Atg8 cleavage of ÄcnaA mutant in the presence of various divalent metal ions.
A. Vacuolar morphology was monitored by fluorescence microscopy using CpyA-GFP as vacuolar lumen marker. The ÄcnaA mutant were grown in liquid MM with indicated concentration of CaCl2 for 60 h. Scale bar represents 5 ìm. 
B, C and D. Fluorescence microscope observation and Western blots were used to examine the cleavage and localization of GFP-Atg8 under the control of the native atg8 promoter (B and C) or the gpdA promoter (D). WT and ÄcnaA were cultured in MM with or without various divalent metal ions (10 mM CaCl2, 10 mM MgCl2, 10 mM ZnCl2, 2 mM FeCl2 and 0.2 mM CuCl2) for 60 h. Scale bar represents 5 ìm.

Figure S5. Growth morphologies of the ÄcnaAÄcchA mutant.
·	DIC images of hyphae grown in liquid MM with or without 10 mM CaCl2 at 37°C for stationary culture 14 h. Scale bar represents 10 ìm.
·	Colony morphologies of WT, ÄcnaA and ÄcnaAÄcchA on solid MM or MM plus 10 mM CaCl2. The 1×10^4^ conidia were inoculated onto solid media for 48 h at 37°C. Statistical significance was determined by Student's t-test. Statistical significance was determined by Student's t-test. ✱✱p < 0.01.

Figure S6. The transcript levels of pmcA/B/C, vcxA/B/C/D/E, and yvcA in the wild-type and ÄcnaA strains grown in MM for 36 h. Statistical significance was determined by Student's t-test. *p < 0.05; **p < 0.01; ns, not significant.

Figure S7. The enriched GO terms of the proteins with increased abundance (A) and decreased abundance (B) in the ÄcnaA mutant compared to the wild-type (fold changes > 1.5). GO annotation was derived from the UniProt-GOA database (http://www.ebi.ac.uk/GOA/).

Figure S8. CchA is dephosphorylated at S55 by calcineurin in vivo. 
Tandem mass spectra of GGSLSNYEK from CchA reveal one unique phosphorylated serine residue, S55, in ÄcnaA. The presence of identified C-terminal (y) and N-terminal (b) product ions are indicated within the peptide sequence. The b and y ions are indicated with red and green colors, respectively.

Table S1. A list of A. fumigatus strains used in this study.
Strains	Genotype and Source	
ZC03/WT	Äku80; pyrG1; AMA1::PgpdA::Cas9::pyr4; from A1160 transformed by FM-6  	
ÄcnaA	ZC03£»ÄcnaA::ptrA	
ÄcnaB	ZC03£»ÄcnaB::ptrA	
cnaAR	Äku80; pyrG1; AMA1::PgpdA::Cas9::pyr4; ÄcnaA::ptrA; cnaA::hph	
cnaBR	Äku80; pyrG1; AMA1::PgpdA::Cas9::pyr4; ÄcnaB::ptrA; cnaB::hph	
ÄcnaA (uridine and uracil auxotroph)	Äku80; pyrG1; ÄcnaA::ptrA; It was generated by deleting FM-6 by 5-FOA under ÄcnaA background.	
WTcyt	Äku80; pyrG1; AMA1::PgpdA::Aeq::pyr4	
ÄcnaAcyt	Äku80; pyrG1; ÄcnaA::ptrA; AMA1::PgpdA::Aeq::pyr4	
cnaARcyt	Äku80; pyrG1; ÄcnaA::ptrA; cnaA::hph; AMA1::PgpdA::Aeq::pyr4	
WTmt	Äku80; pyrG1; AMA1::PgpdA::mt-Aeq::pyr4	
ÄcnaAmt	Äku80; pyrG1; ÄcnaA::ptrA;AMA1::PgpdA::mt-Aeq::pyr4	
cnaARmt	Äku80; pyrG1; ÄcnaA::ptrA; cnaA::hph; AMA1::PgpdA::mt-Aeq::pyr4	
WTcpyA-gfp	Äku80; pyrG1; AMA1::PgpdA::cpyA-GFP::pyr4	
gfp-pmcA
cpyA-rfp	Äku80; pyrG1; PgpdA::gfp-pmcA::ptrA; AMA1::PgpdA::cpyA-RFP::pyr4	
WTvac	Äku80; pyrG1; AMA1::PgpdA::cpyA-Aeq::pyr4	
OE::pmcAvac	Äku80; pyrG1; AMA1::PgpdA::cpyA-Aeq::pyr4; PgpdA::pmcA::hph	
OE::pmcBvac	Äku80; pyrG1; AMA1::PgpdA::cpyA-Aeq::pyr4; PgpdA::pmcB::hph	
ÄcnaAvac	Äku80; pyrG1; ÄcnaA::ptrA; AMA1::PgpdA::cpyA-Aeq::pyr4	
cnaARvac	Äku80; pyrG1; ÄcnaA::ptrA; cnaA::hph; AMA1::PgpdA::cpyA-Aeq::pyr4	
WTrfp-H2A	Äku80; pyrG1; AMA1::PgpdA::RFP-H2A::pyr4	
ÄcnaArfp-H2A	Äku80; pyrG1; ÄcnaA::ptrA; AMA1::PgpdA::RFP-H2A::pyr4	
WTerg11A-gfp	Äku80; pyrG1; AMA1::PgpdA::erg11A-GFP::pyr4	
ÄcnaAerg11A-gfp	Äku80; pyrG1; ÄcnaA::ptrA; AMA1::PgpdA::erg11A-GFP::pyr4	
WTrfp-ph-osbp	Äku80; pyrG1; AMA1::PgpdA::RFP-PHOSBP::pyr4	
ÄcnaArfp-ph-osbp	Äku80; pyrG1; ÄcnaA::ptrA;AMA1::PgpdA::RFP-PHOSBP::pyr4	
WTmrsA-rfp	Äku80; pyrG1; AMA1::PgpdA::MrsA-RFP::pyr4	
ÄcnaAmrsA-rfp	Äku80; pyrG1; ÄcnaA::ptrA; AMA1::PgpdA::MrsA-RFP::pyr4	
WTPgpdA-gfp-atg8	Äku80; pyrG1; AMA1::PgpdA::GFP-Atg8::pyr4	
ÄcnaAPgpdA-gfp-atg8	Äku80; pyrG1; ÄcnaA::ptrA; AMA1::PgpdA::GFP-Atg8::pyr4	
WTPgpdA-gfp-atg8,cpyA-rfp	Äku80; pyrG1; AMA1::PgpdA::CpyA-rfp::PgpdA::GFP-Atg8::pyr4	
ÄcnaAPgpdA-gfp-atg8,cpyA-rfp	Äku80; pyrG1; ÄcnaA::ptrA; AMA1::PgpdA::CpyA-rfp::PgpdA::GFP-Atg8::pyr4	
WTPatg8-gfp-atg8	Äku80; pyrG1; AMA1::Patg8::GFP-Atg8::pyr4	
ÄcnaAPatg8-gfp-atg8	Äku80; pyrG1; ÄcnaA::ptrA; AMA1::Patg8::GFP-Atg8::pyr4	
ÄcnaAÄatg2	ZC03; ÄcnaA::ptrA; Äatg2::hph	
ÄcnaAÄatg2PgpdA-gfp-atg8,cpyA-rfp	Äku80; pyrG1; ÄcnaA::ptrA; Äatg2::hph; AMA1::PgpdA::CpyA-rfp::PgpdA::GFP-Atg8::pyr4	
ÄcnaAÄatg2PgpdA-gfp-atg8	Äku80; pyrG1; ÄcnaA::ptrA; Äatg2::hph; AMA1::PgpdA::GFP-Atg8::pyr4	
ÄcnaAÄcchA	ZC03; ÄcnaA::ptrA; ÄcchA::hph	
ÄcnaAÄcchAPgpdA-gfp-atg8,cpyA-rfp	Äku80; pyrG1;ÄcnaA::ptrA; ÄcchA::hph; AMA1::PgpdA::CpyA-rfp::PgpdA::GFP-Atg8::pyr4	
ÄcnaAÄcchAPgpdA-gfp-atg8	Äku80; pyrG1; ÄcnaA::ptrA; ÄcchA::hph; AMA1::PgpdA::GFP-Atg8::pyr4	
ÄcnaAÄcchArfp-H2A	Äku80; pyrG1; ÄcnaA::ptrA; ÄcchA::hph; AMA1::PgpdA::RFP-H2A::pyr4	
ÄcnaAÄcchArfp-ph-osbp	Äku80; pyrG1; ÄcnaA::ptrA; ÄcchA::hph; AMA1::PgpdA::RFP-PHOSBP::pyr4	
ÄcnaAÄcchAvac	Äku80; pyrG1; ÄcnaA::ptrA; ÄcchA::hph; AMA1::PgpdA::cpyA-Aeq::pyr4	
WTluc	Äku80; pyrG1; PgpdA::Luc::pyr4	
ÄcnaAluc	Äku80; pyrG1; ÄcnaA::ptrA; PgpdA::Luc::pyr4	
ÄcnaAÄcchAluc	Äku80; pyrG1; ÄcchA::hph; ÄcnaA::ptrA; PgpdA::Luc::pyr4	
ÄcnaAOE::pmcA	ZC03; ÄcnaA::ptrA; PgpdA::pmcA::hph	
ÄcnaAOE::pmcAvac	Äku80; pyrG1; ÄcnaA::ptrA£»AMA1::PgpdA::cpyA-Aeq::pyr4; PgpdA::pmcA::hph	
ÄcnaA OE::pmcAPgpdA-gfp-atg8,cpyA-rfp	Äku80; pyrG1; ÄcnaA::ptrA£»AMA1::PgpdA::CpyA-rfp::PgpdA::GFP-Atg8::pyr4; PgpdA::pmcA::hph	


Table S2. All the primers and primer annotations.
Name	Sequence	Intention	
T7-cnaA-sgRNA5-F	TAATACGACTCACTATAGGGCGCAAGATGGATCAAGCACGTTTTAGAGCTAGAAATAGC	for the DNA template of cnaA-sgRNA3(RNA); for deleting cnaA	
cnaA-ptrA-F	GACGTAATATTCCTTAGTTCACACTGCGCAAGCTAGGAGATCGTCCGCCGATG	for the repair template of deleting cnaA	
cnaA-ptrA-R	CAGATCTTCAGGGCCCAGAGATATAAAACGCAGCCTAGATGGCCTCTTGCATC	for the repair template of deleting cnaA	
cnaA-seq-F	AACCATTATCCACCGGAGGATTG	diagnostic primer for ÄcnaA	
cnaA-seq-R	TGATTCCTTCGGCGCCAAGCATC	diagnostic primer for ÄcnaA	
hph-F	GAATTCCCTTGTATCTCTAC	for hph	
hph-R	TCGAGTGGAGATGTGGAGTG	for hph	
cnaA-not1-F	AAGGGCAATTCGCGGCCCTGATTCTGATAGTAGCACAGCG	for the revertant of cnaA mutant	
cnaA-not1-R	CGAATTGAATTTAGCGGCCCGCATGACAGGTTGAATGCAG	for the revertant of cnaA mutant	
T7-cnaB-sgRNA-F	TAATACGACTCACTATAGGGTTGTTTCAAGGAGAGGACGTTTTAGAGCTAGAAATAGCA	for the DNA template of cnaB-sgRNA	
cnaB-ptrA-F	ACTCCTCAACCACACACGACCATTTACCAACGCCTAGATGGCCTCTTGCATC	for the repair template of deleting cnaB ORF	
cnaB-ptrA-R	TCAATCACAATCGGTCAGTCCTCTCCTGTCCTAGGAGATCGTCCGCCGATG	for the repair template of deleting cnaB ORF	
cnaB-seq-F	CTCGCCTCTACTGCACGTTC	diagnostic primer for ÄcnaB	
cnaB-seq-R	AACGCCAGGTAGCAAGACTC	diagnostic primer for ÄcnaB	
cnaB-notI-F	AAGGGCCAATTCGCGGCCGTTTCCACCTGTTGTAG	for the revertant of cnaB mutant	
cnaB-notI-R	CGAATTGAATTTAGCGGCCCCTTGGATAGAAGAGATCTGCC	for the revertant of cnaB mutant	
Ama1-BamHI-gpd-F	CGGTTATGCCGTATGGATCCGCATGCGGAGAGACGGACG	for gpdA promoter	
Ama1-BamHI-trpC-R	AATCAGCCTAGCTAGGATCCCATGCATTGCAGATGAGCTG	for trpC terminator	
ClaI-cpyA-F£º	CTTTAATCAAGCTTATCGATATGAGAGTTCTTCCAGCTACA	for cpyA ORF	
ClaI-cpyA-R£º	TCGAGGTCGACGGTATCGATTTAGAACCATTCACCACCAAGC	for cpyA ORF	
CpyA-linker-R	GGCACCGGCTCCAGCGCCTGCACCAGCTCCGAACCATTCACCACCAAGCC	for cpyA	
Linker-GFP/RFP-F	GGAGCTGGTGCAGGCGCTG	for GFP/RFP	
Ama1-BamHI-GFP-R	TCAGCCTAGCTAGGATCCTTATTTGTATAGTTCATCCATGC	for GFP	
Ama1-BamHI-RFP-R	AATCAGCCTAGCTAGGATCCTTAGGCGCCGGTGGAGTGGC	for RFP	
Linker-Aeq-F	CAGGCGCTGGAGCCGGTGCCATGACCTCCAAGCAGTACT	for Aeq	
ClaI-pmcA-F	CTTTAATCAAGCTTATCGATATGTCATCGAATCCAAACCAA	for pmcA ORF	
ClaI-pmcA-R	TCGAGGTCGACGGTATCGATCTAGCTTTGTCGCGACTGGC	for pmcA ORF	
RT-pmcA-F	CGATCTCGCTCTAACTCTGC	RT primer for pmcA	
RT-pmcA-R	TAATCTCCGACGAGACGCTC	RT primer for pmcA	
ClaI-pmcB-F	CTTTAATCAAGCTTATCGATATGCTCAATCCCAAGTCGCTC	for pmcB ORF	
ClaI-pmcB-R	CGAGGTCGACGGTATCGATTTAAGAGCTCGTAATTGTAATGGGA	for pmcB ORF	
RT-pmcB-F	ATGTGACTCGCCTGGACGCTG	RT primer for pmcB	
RT-pmcB-R	CAGGCCACATCATAGACGAG	RT primer for pmcB	
Erg11A-R	CTTGGATGTGTTTTTCGACCGCTTC	for PgpdA-Erg11A	
Erg11A-GFP-F	CGGTCGAAAAACACATCCAAGGGAGCTGGTGCAGGCGCTGG	for GFP	
Ama1-BamHI-MrsA-F	AATCAGCCTAGCTAGGATCCTTCGTAAACCTACAGCTCCTG 	for MrsA	
MrsA-R	CTCCTGGCGTTTGAAGTAG	for MrsA	
MrsA-RFP	CTACTTCAAACGCCAGGAGGGAGCTGGTGCAGGCGCTG	for RFP	
Gpd-R	ATCGATAAGCTTGATTAAAGGTT	for gpdA promoter	
GpdA-RFP-F	GAACCTTTAATCAAGCTTATCGATATGGCCTCCTCCGAGGACGTCA	For RFP-PHOSBP	
Ama1-BamHI-phosbp-R	AATCAGCCTAGCTAGGATCC TCACGAATTCTTCTTCACAGC
	For RFP-PHOSBP	
Gpd-GFP-F	TTTAATCAAGCTTATCGATATGAGTAAAGGAGAAGAACTTTTCAC	for GFP-Atg8	
Ama1-BamHI-Atg8-R	AATCAGCCTAGCTAGGATCCTCAGCAGTCACCGAAAGTGTTC	for GFP-Atg8	
Ama1-Bam1-atg8(p)-F	CGGTTATGCCGTATGGATCCCAGCCCACCGTGTAGTAGAG
	For atg8 promoter	
Atg8-promoter-R	CTTGATAGATAAGGGCGGATAACG
	For atg8 promoter	
Atg8(p)-GFP-F	GTTATCCGCCCTTATCTATCAAGATGAGTAAAGGAGAAGAACTTTTCAC
	for GFP-Atg8	
TAA-RFP-R	TTAGGCGCCGGTGGAGTGGC
	primer for PgpdA-CpyA-RFP	
RFP-gpdA-F	 GCCACTCCACCGGCGCCTAA
GCATGCGGAGAGACGGACG 	Primer for PgpdA-GFP-Atg8
	
			
T7-atg2-F	TAATACGACTCACTATAGGGCAAGAAGTAAGCCATCTGTTTTAGAGCTAGAAATAGCA
	for the DNA template of atg2-sgRNA1, for deleting atg2	
Atg2-hph-F	GAGCCGCTGGTTGGCTCGTTTCGCCCGAGGAATTCCCTTGTATCTCTACACAC
	for the repair template of deleting atg2	
Atg2-hph-R	GAAGGCTCGCTTGACTTTGGCTGCACGGGTGTAGTCTCGAGTGGAGATGTGGAGTGGGC
	or the repair template of deleting atg2	
Atg2-seq-F	TGATCATCGCTCGCTATCTGC	diagnostic primer for Äatg2	
Atg2-seq-R	CTGTGTCTCCAAGATTGTCG
	diagnostic primer for Äatg2	
T7-cchA-sgRNA1-F	TAATACGACTCACTATAGGGTCTGCTTTGCAGCGGGCGTTTTAGAGCTAGAAATAGCA	for the DNA template of cchA-sgRNA1; for deleting cchA	
cchA-hph-F	TACATTCGGCTAGCGCATTATACCTGCCTACGGAATTCCCTTGTATCTCTACACAC	for the repair template of deleting cchA	
cchA-hph-R	TGCGACATCGACTTCATCATCGAACCCGCCTCGAGTGGAGATGTGGAGTGGGC	for the repair template of deleting cchA	
cchA-seq-F	ACAGCTGCAGTCTGCAATGTC	diagnostic primer for ÄcchA	
cchA-seq-R	GATTGTTGTACATCCTCCACGC	diagnostic primer for ÄcchA	
NOTI-gfp-F	CGAATTGAATTTAGCGGCCGGAGCTGGTGCAGGCGCTGG	for GFP	
NOTI-gfp-R	AAGGGCAATTCGCGGCCCTATTATTTGTATAGTTCATCCATGCC	for GFP	
NotI-PgpdA-F	AAGGGCCAATTCGCGGCCGCGCATGCGGAGAGACGGACG	for gpdA promoter	
NotI-TtrpC-R	AATTGAATTTAAGCGGCCGCCATGCATTGCAGATGAGCTG	for trpC terminator	
ptrA-F	CATGGCAGACACTGAAGCAAC	for ptrA	
ptrA-R	TAGCCTAGATGGCCTCTTGCATC	for ptrA	
zero-F	ACTAGTCCTGCAGGTTTAAACGAA	linearizating p-zero-ptrA	
zero-R	CCCTTTAGTGAGGGTTAATTCTG	linearizating p-zero-ptrA	
zero-PgpdA-F	TTTAAACCTGCAGGACTAGTGCATGCGGAGAGACGGACG	for gpdA promoter	
PgpdA-R	ATCGATAAGCTTGATTAAAGGTTCT	for gpdA promoter	
PgpdA-GFP-F	AGAACCTTTAATCAAGCTTATCGATATGAGTAAAGGAGAAGAACTTTTCAC	for GFP	
zero-GFP-R	AATTAACCCTCACTAAAGGGCTGGATCTCGGAGATTTTGTATAG	for GFP	
CchA-GFP-R	TTGTGGCTATTCGAAGCCATCTGGATCTCGGAGATTTTGTATAG	for PgpdA-GFP	
CchA-F	ATGGCTTCGAATAGCCACAAC	for CchAN300	
Ama1-BamHI-CchAN300-R	AATCAGCCTAGCTAGGATCCTTATTGATCATCAGACATTCCGACAGAC	for CchAN300	
ClaI-Luc-F	CTTTAATCAAGCTTATCGATATGGAAGATGCCAAAAACATTAAG	for luc	
ClaI-Luc-R	TCGAGGTCGACGGTATCGATTTCTTGGCCTTAATGAGAATCTCG	for luc	
RT-pmcC-F	CTCTGGCCTTAGCAACCGAC	RT primer for pmcC	
RT-pmcC-R	TGAAGATGACGGTGTCGAGC	RT primer for pmcC	
RT-yvcA-F	GCGATCTTCGCTCTGAAGAC	RT primer for yvcA	
RT-yvcA-R	ATCCTCTTCTCCCTGACGAC	RT primer for yvcA	
RT-vcxA-F	GATGACTCTCAACTTCCACATC	RT primer for vcxA	
RT-vcxA-R	GCATCGCCAGTAGGATCATC	RT primer for vcxA	
RT-vcxB-F	GTCATGTTACTGGTGTCAACTGC	RT primer for vcxB	
RT-vcxB-R	TGTCTTTGTCCATGCACCAGC	RT primer for vcxB	
RT-vcxC-F	CACCAAGCTCACTCTGACC	RT primer for vcxC	
RT-vcxC-R	GATTTGATTGTCCCAGGCTTCGTC	RT primer for vcxC	
RT-vcxD-F	GAACCCATCAACGTGTCCAAC	RT primer for vcxD	
RT-vcxD-R	GTAATTCAGTGCAATTCCAACTGG	RT primer for vcxD	
RT-vcxE-F	GTTGGTTCCATTGACGCTCTTAC	RT primer for vcxE	
RT-vcxE-R	CCATCGGCAATGAGATAGTTCAC	RT primer for vcxE	
